# Ultrasound (US)-guided percutaneous thrombin injection for stoma-site bleeding after PEG tube insertion: a case series and review of the literature

**DOI:** 10.1186/s42155-024-00432-y

**Published:** 2024-02-20

**Authors:** Aws Alfahad, Rawan Alhalabi

**Affiliations:** American Hospital Dubai, Dubai, UAE

**Keywords:** GI bleeding, Anticoagulants, Ultrasound, Thrombin, Percutaneous Thrombin Injections PTI, Interventional radiology

## Abstract

**Background:**

Post-gastrostomy bleeding sequelae are acknowledged, with reported approaches focusing on conservative measures or surgical repair. Nonetheless, Percutaneous Thrombin Injections (PTI) role in PEG-site-related bleeding remains underexplored. PTI under ultrasound guidance is an advocated management strategy for stoma-site bleeding following gastrostomy in high-risk patients, particularly those on direct oral anticoagulants.

**Case presentation:**

This study presents three cases with multiple comorbidities who underwent PTI. Resulting in immediate resolution of bleeding, no systemic\local effect, and no reported complications or rebleeding after a 3–6-month follow-up.

**Conclusion:**

The findings highlight the safety, direct complete resolution, and absence of sequelae associated with PTI, suggesting its potential as a promising technique in managing PEG stoma-related bleeding.

**Supplementary Information:**

The online version contains supplementary material available at 10.1186/s42155-024-00432-y.

## Background

Hemostasis represents the process of clot formation initiated by vascular endothelial damage following injuries. Thrombin plays a major role in the coagulation cascade by transforming circulating fibrinogen into fibrin, culminating in the formation of a stable clot that effectively prevents further bleeding [1, 2].

Direct Oral Anticoagulants (DOACs), inhibitors of thrombin, and antiplatelets are indicated in various clinical scenarios like Cerebrovascular Accident (CVA), Myocardial Infarction (MI), Atrial Fibrillation (AF), and Deep Venous Thromboembolism (DVT) [1, 3]. While these agents demonstrate remarkable efficacy, they come with a notable risk of significant bleeding. Elevated susceptibility to major hemorrhages is correlated with advanced age and comorbidities [4]. Gastrointestinal (GI) bleeding is a notable concern [4–6]. Representing nearly half of the major bleeds with carrying a 10% risk of morbidity or mortality [6].

This report details multiple cases involving elderlies with comorbidities who underwent percutaneous thrombin injections (PTI) under ultrasound (US) guidance. This intervention served as a management or prophylactic measure for stoma-site bleeding following gastrostomy in patients taking DOAC or antiplatelet.

### Technique

Mainly Percutaneous Endoscopic Gastrostomy (PEG) tube insertion is done by the interventional radiologists (IR) at our facility.

After the gastrostomy, all our patients receive intravenous antibiotics and proton pump inhibitors for at least 3 days to decrease the risk of peritonitis and stress gastritis.

**Fig. 1:**
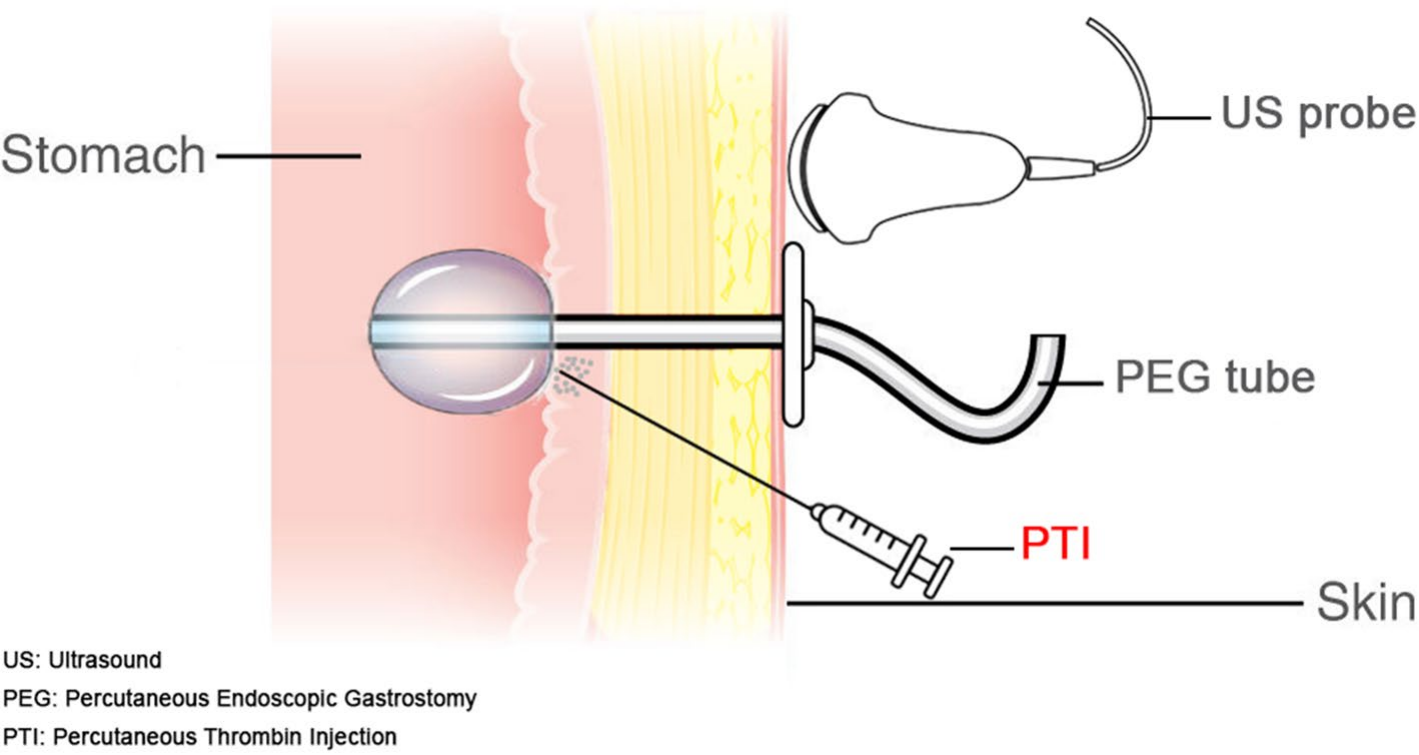
Illustration of PTI technique

**Fig. 2:**
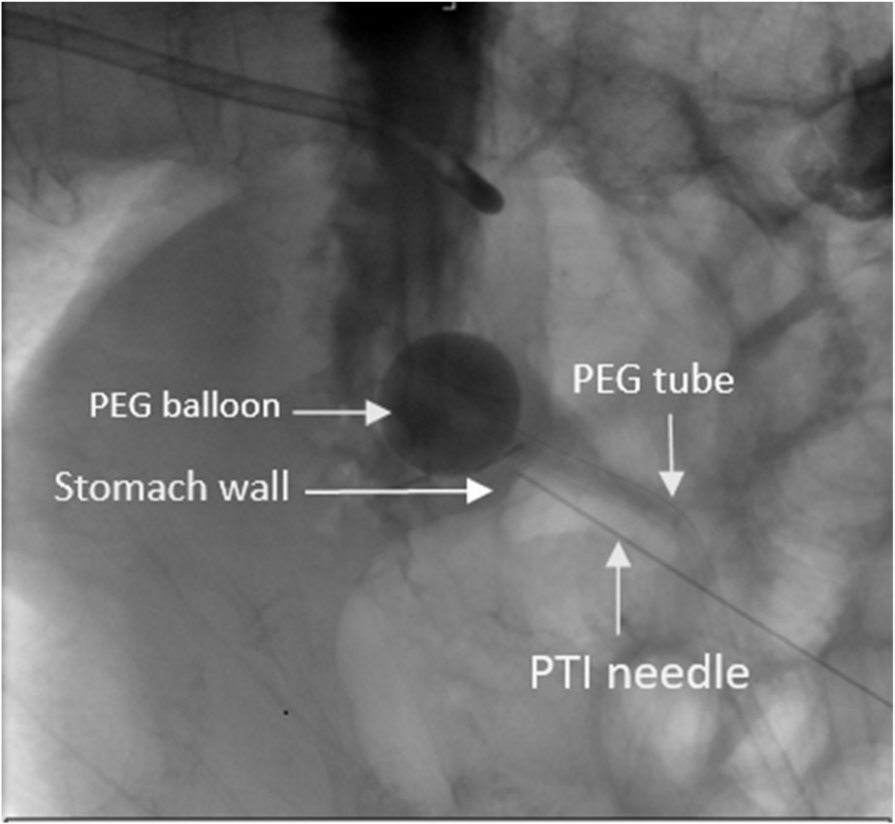
Fluoroscopy image demonstrating the needle site

The technique of PTI relies primarily on identifying the PEG tube orifice (refer to Figs. 1, 2, and the attached video (link: PTI video).

Initially, non-enhanced ultrasound guidance was engaged to assess the stomach, confirm the PEG position, and guide the needle. A 20 mL solution of 1% lidocaine with epinephrine was locally administered, filtering through the abdominal wall down to the parastomal gastric wall. Following this, thrombin is injected into the stomach wall, encircling all sides of the parastomal region while avoiding the balloon.

**Fig. 3:**
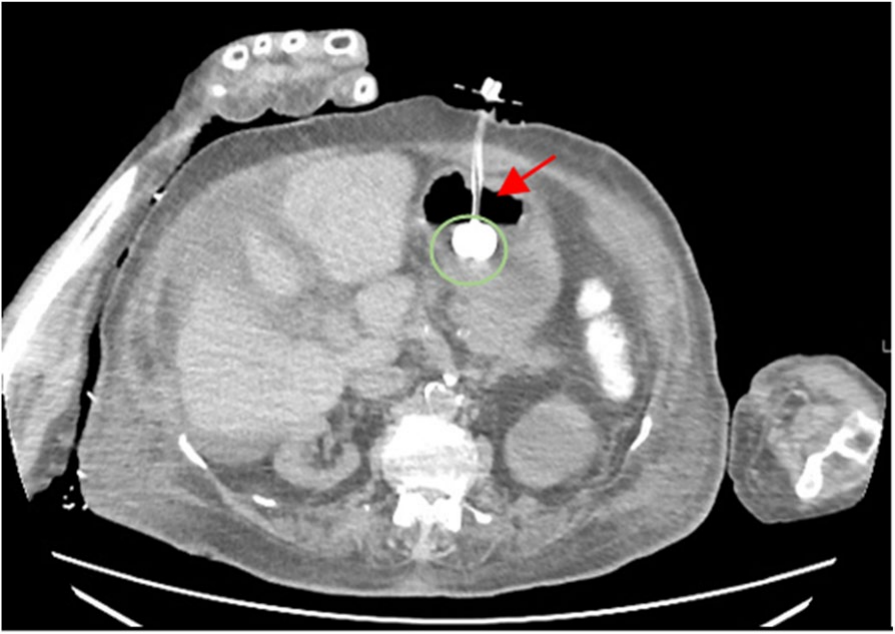
A non-enhanced CT scan of the PEG tube three days post-enjection, showcasing a patent ballon (green circle), a clear stomach wall devoid of necrosis or hematoma, and an absence of any bleeding (red arrow)

**Fig. 4:**
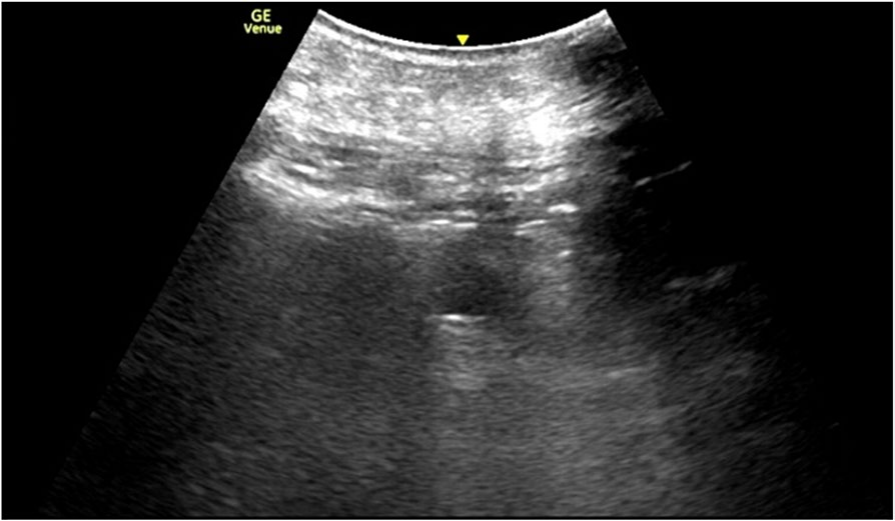
US post PTI revealing patent PEG balloon and no hemorrhage

Figures 3 and 4 show a clear patent PEG balloon with a non-complicated site

We suggest a 2500 International Units (IU) dose for all patients. We have observed its effectiveness in all cases, irrespective of weight, the extent of bleeding, or the size of the PEG tube.

## Case presentation

### Case 1

A 76-year-old male with a medical history including atrial fibrillation, chronic kidney disease, diabetes mellitus, hypertension, and congestive heart failure, was admitted to our hospital for intravenous antibiotics to treat a resistant urinary tract infection. The patient was on Apixaban.

Due to poor oral intake, Percutaneous Endoscopic Gastrostomy (PEG) placement was deemed necessary. Post-procedure, significant bleeding occurred from the stomach wall through the PEG orifice over the first day leading to hypotension and blood transfusion. Promptly, 2500 IU of thrombin were administered, resulting in immediate cessation of bleeding. The patient, who remains on DOAC, has shown no signs of rebleeding in the six months following the intervention.

### Case 2

A 38-year-old male patient was admitted to our hospital for further management of recurrent cardiovascular accidents that resulted in dense left-side hemiplegia and weakness on the right side associated with dysphagia. The patient has a medical history that includes diabetes mellitus, dyslipidemia, and hypertension, with a significant history of heavy smoking and nicotine abuse. He is on clopidogrel, aspirin, and low molecular-weight heparin.

At our facility, he underwent gastrostomy which was complicated with site bleeding, leading to symptoms of melena and hematemesis resulting in hypovolemic shock and ICU admission. He had persistent GI bleeding for 2 days. An abdominal CT scan with contrast showed no active intraabdominal bleeding. Following the failed conservative management, a therapeutic intervention involved the administration of 2500 IU of thrombin at the parastomal region under US guidance which directly resolved the bleeding. The patient was also initiated on heparin for deep vein thrombosis (DVT) prophylaxis and subsequently discharged with aspirin. Upon follow-up at 6 months, there were no reports of rebleeding or systematic complications.

### Case 3

A 100-year-old lady visited the hospital complaining of poor oral intake persistent for 10 days. She has multiple comorbidities including dementia, atrial fibrillation, chronic kidney disease, hyperkalemia, and limited mobility. Accordingly, she was on Apixaban.

On examination, the patient was severely dehydrated.

The poor intake was attributed to the presence of oral ulcers and dental pain. It was decided to perform a gastrostomy procedure. To minimize the risk of post-procedure bleeding, 2500 IU of thrombin was injected at the gastrostomy insertion site under US guidance.

During a 3-month follow-up, there were no signs of bleeding although the patient is on continuous anticoagulation therapy.

## Conclusion

The Percutaneous Endoscopic Gastrostomy (PEG) tube serves as a crucial enteral feeding method for managing critically ill patients, ensuring direct delivery of nutrition into the Gastrointestinal Tract (GIT) [7–9]. While generally considered safe, complications can pose serious risks which can lead to unfavorable sequelae or death. GI bleeding is an outstanding concern that was reported in up to 2.5–3% of the cases [7, 8, 10].

In our comprehensive literature search across PubMed, Medline, Scopus, and Google Scholar, the existing approaches for managing post-PEG site bleeding predominantly involve conservative measures, transcatheter arterial embolization, adjustments in anticoagulant medications, endoscopic interventions, or surgical repair for life-threatening situations [7–10].

However, there is a gap in research regarding the use of PTI for managing stoma-site bleeding, particularly in high-risk patients. Nonetheless, Previous reports have highlighted successful PTI applications in pseudoaneurysms and hemoperitoneum related to paracentesis [11–16].

For comorbid patients undergoing emergency procedures under general anesthesia, the risks are substantial, with a mortality rate of 18% and a major complication rate of 65% [17, 18]. Thus, the decision to perform endoscopy or surgical intervention in such cases is fraught with significant risks. Furthermore, a retrospective study revealed that 22% of high-risk patients undergoing endoscopic procedures experienced deterioration leading to death, primarily attributed to pneumoperitoneum resulting from gas inflation, with added challenges in the presence of a stomach stoma [19]. In terms of Transcatheter arterial embolization (TAE), it is found to be associated with a 27.8% rebleeding rate within 30 days and an 11% mortality rate due to rebleeding [20], which is also a considerable risk.

In the context of our intervention, the vasoconstrictive effect of epinephrine plays a fundamental role in effectively controlling bleeding [21]. We strongly emphasize that this contribution has significantly influenced the success of the PTI procedure.

Our study advocates that PTI with epinephrine, applied prophylactically or as a treatment under local anesthesia, finds wide acceptance for managing PEG-site bleeding, particularly when conservative therapy fails. It emerges as a preferable alternative to anesthesia-requiring interventions in high-risk patients, demonstrating a safety profile. However, potential limitations in cases of upper GI bleeding with diverse bleeding sites necessitate further studies for validation of success rates and complications.

We recommend proactive administration for high-risk patients, including those with chronic kidney disease, cirrhosis, elderly individuals with limited cardiac reserve, patients needing prompt resumption of antiplatelet or anticoagulant therapy after Gastrostomy, and those with a history of previous gastric bleeding.

Importantly, reported cases and our findings consistently show no systematic/local complications or failures, highlighting the direct and effective complete resolution of bleeding as a considerable outcome. Moreover, the accessibility and flexibility of ultrasound significantly ease the implementation of PTI in emergency scenarios or during combined IR procedures, underscoring the critical value of safety, immediate resolution, and the absence of complications in the study. PTI thus arises as a promising possibility in the management of PEG tube-related bleeding.

While the occurrence of bleeding complications after gastrostomy is relatively low, addressing these complications is crucial due to their potential for severe consequences, including fatality. Emphasizing the importance of care and prompt bleeding cessation in this study becomes a principal in establishing PTI as a viable and effective therapeutic approach for PEG tube bleeding, especially in high-risk patients.

### Supplementary Information

**Additional file 1**.

## Data Availability

Not applicable.

## Abbreviations

PTI: Percutaneous thrombin injection
US: Ultrasound
DOACs: Direct oral anticoagulants
CVA: Cerebrovascular accident
MI: Myocardial infarction
AF: Atrial fibrillation
DVT: Deep venous thromboembolism
GI: Gastrointestinal
PEG: Percutaneous endoscopic gastrostomy
IU: International units
GIT: Gastrointestinal tract
TAE: Transcatheter arterial embolization
